# Acupuncture as an intervention to reduce alcohol dependency: a systematic review and meta-analysis

**DOI:** 10.1186/s13020-016-0119-4

**Published:** 2016-12-15

**Authors:** Charlotte Southern, Charlie Lloyd, Jianping Liu, Congcong Wang, Tingting Zhang, Martin Bland, Hugh MacPherson

**Affiliations:** 1 Department of Health Sciences, University of York, York, UK; 2 Centre for Evidence-Based Chinese Medicine, Beijing University of Chinese Medicine, Beijing, China

## Abstract

**Background:**

Acupuncture has been widely used as a treatment for alcohol dependence. An updated and rigorously conducted systematic review is needed to establish the extent and quality of the evidence on the effectiveness of acupuncture as an intervention for reducing alcohol dependence. This review aimed to ascertain the effectiveness of acupuncture for reducing alcohol dependence as assessed by changes in either craving or withdrawal symptoms.

**Methods:**

In this systematic review, a search strategy was designed to identify randomised controlled trials (RCTs) published in either the English or Chinese literature, with a priori eligibility criteria. The following English language databases were searched from inception until June 2015: AMED, Cochrane Library, EMBASE, MEDLINE, PsycINFO, and PubMed; and the following Chinese language databases were similarly searched: CNKI, Sino-med, VIP, and WanFang. Methodological quality of identified RCTs was assessed using the Jadad Scale and the Cochrane Risk of Bias tool.

**Results:**

Fifteen RCTs were included in this review, comprising 1378 participants. The majority of the RCTs were rated as having poor methodological rigour. A statistically significant effect was found in the two primary analyses: acupuncture reduced alcohol craving compared with all controls (SMD = −1.24, 95% CI = −1.96 to −0.51); and acupuncture reduced alcohol withdrawal symptoms compared with all controls (SMD = −0.50, 95% CI = −0.83 to −0.17). In secondary analyses: acupuncture reduced craving compared with sham acupuncture (SMD = −1.00, 95% CI = −1.79 to −0.21); acupuncture reduced craving compared with controls in RCTs conducted in Western countries (SMD = −1.15, 95% CI = −2.12 to −0.18); and acupuncture reduced craving compared with controls in RCTs with only male participants (SMD = −1.68, 95% CI = −2.62 to −0.75).

**Conclusion:**

This study showed that acupuncture was potentially effective in reducing alcohol craving and withdrawal symptoms and could be considered as an additional treatment choice and/or referral option within national healthcare systems.

**Electronic supplementary material:**

The online version of this article (doi:10.1186/s13020-016-0119-4) contains supplementary material, which is available to authorized users.

## Background

Approximately 3.3 million deaths worldwide are attributed to alcohol dependency per year [1]. The prevalence of alcohol dependency in the UK in 2010 was estimated at 5.9% of the population (8.7% of men and 3.2% of women), compared with 4% in Europe [1, 2]. A dependent drinker who stops drinking will experience alcohol withdrawal syndrome; this is a distressing and life-threatening condition with symptoms that range in severity, including tremors, agitation, paroxysmal sweats, fever, nausea, and seizures [3]. These symptoms typically occur within the first 24 h of stopping drinking and can last a number of weeks [4].

In England, treatment for alcohol dependency is received by a minority (6%) of an estimated 1 million people aged 16–65 years who are alcohol dependent [2]. The UK National Institute for Health and Care Excellence (NICE) reports that non-pharmacological treatments are an important therapeutic option for people with alcohol-related problems, and that acupuncture is valued by service users with alcohol-related problems [2]; however, NICE acknowledges that the evidence base for the effectiveness of acupuncture is weak [2].

A preliminary search of the field identified two reviews specifically related to acupuncture as an intervention for alcohol disorders [5, 6]. The review by Kunz et al. [5] included 14 studies investigating the effectiveness of auricular acupuncture in the treatment of withdrawal from substances (opiate, cocaine, and alcohol). The authors decided not to conduct a meta-analysis owing to potential systematic and selection biases. The findings for the review were inconclusive and the effectiveness of auricular acupuncture as an intervention for withdrawal was not determined. The included studies lacked rigorous methodology, resulting in reduced internal validity. In this review, Chinese language studies were excluded.

A subsequent review by Cho and Whang [6] included 11 studies and did not limit acupuncture techniques to auricular acupuncture [6]. A meta-analysis for treatment completion rates identified no statistically significant difference between acupuncture and either sham or no acupuncture groups. The results were equivocal and the included studies lacked rigorous methodology. However, the review included languages other than English, which increased its robustness.

An updated review is needed, using rigorous review methods. In the present review, we refined the search for acupuncture as a treatment for alcohol craving and withdrawal symptoms in alcohol-dependent individuals. We included randomised controlled studies (RCTs) published in both the English and Chinese literature, and conduct meta-analyses on the main outcome measures. Therefore, the present review expands on the existing research in this area to provide fresh and relevant evidence from all RCTs to establish whether acupuncture is effective in reducing alcohol craving and withdrawal symptoms.

## Methods

### Eligibility of studies for this systematic review

Inclusion and exclusion criteria were pre-specified (Table 1).

**Table 1:** Defined inclusion and exclusion criteria

Population	Alcohol dependents, inpatients and outpatients. Animal studies excluded
Intervention	Acupuncture where the needle punctures the skin surface at acupuncture points; either auricular or body
Control	Sham acupuncture or treatment as usual or other treatment
Outcomes	Primary measures: alcohol craving and alcohol-withdrawal symptoms Secondary: adverse effects
Study design	RCTs comparing acupuncture to a control. Restricted to English and Chinese language publications

### Search method

The following English language databases were searched up to June 2015: AMED (OvidSP) (from 1985), the Cochrane Library, EMBASE (OVID) (from 1946), MEDLINE (OVID) (from 1946), PsycINFO (from 1987), and PubMed (from 1970); and the following Chinese language database were searched up to June 2015: CNKI (from 1994), Sino-med (from 1960), VIP (from 1989), and WanFang (from 1998). Table 2 presents the keywords used, and Appendix S1 (Additional file 1) provides the MeSH terms and keywords used in the Medline (OVID) search.

**Table 2:** Key terms (or nearest appropriate Chinese equivalent)

PICOS	Key search terms
P	Alcohol abuse; alcoholism; alcohol drinking pattern; alcohol related disorders; binge drink$; drinking behaviour; alcohol dependence; alcohol withdrawal; alcohol dependent; alcohol abstinence; alcohol addiction; alcohol misuse; alcoholism treatment; alcoholic beverage$; alcohol intoxication; alcohol withdrawal delirium; human; inpatients; outpatients; rehab$; primary care; secondary care
I	Acupuncture; electroacupuncture; meridian; ear acupuncture; auricular acupuncture; body acupuncture; traditional acupuncture; medical acupuncture; traditional Chinese medicine; alternative medicine
C	Sham acupuncture; placebo acupuncture; placebo needles; treatment as usual; conventional medicine; counselling
O	Withdrawal; alcohol withdrawal; craving$
S	Randomised control trials; RCT$; random sampling; experimental design

### Study selection

CS screened the Western databases; titles and abstracts were analysed to exclude irrelevant and duplicate studies. CW screened the Chinese literature using the same criteria. All relevant studies were retrieved as full reports for detailed evaluation. Any study that did not satisfy the inclusion criteria was excluded.

### Data extraction

Data were extracted by CS on study design, participant characteristics, results, and statistical information. Translations of the full Chinese reports were undertaken by TZ at the University of York; these translations were clarified by CS, with further clarification from CW.

### Quality assessment

The included RCTs were assessed for bias and scored using the Cochrane Risk of Bias tool [7], and for methodological quality using the Jadad Scale [8].

### Narrative synthesis

A narrative synthesis was performed to provide a basis for assessing each RCTs contribution to the research question in terms of interpretation, synthesis, and triangulation regarding the quantitative data.

### Quantitative synthesis

Meta-analyses produced the overall effect estimates for the two primary outcomes related to craving and withdrawal symptoms. Continuous outcomes (Visual Analogue Scale and Likert-scaled options used to assess craving and Alcohol Withdrawal Syndrome Scale, the Mainz Alcohol Withdrawal Scale, and the Clinical Institute Withdrawal Assessment for withdrawal) were presented as the standardised mean difference (SMD) with 95% confidence intervals (CI). As each RCT did not use the same acupuncture technique, we could not assume that acupuncture in each RCT was estimating the same effect, therefore we used a random-effects model for meta-analyses. For RCTs containing either two acupuncture groups or two sham acupuncture groups, both arms were combined for the analyses. Analyses of data on craving and withdrawal outcomes at the end of the intervention phase were performed using Comprehensive Meta-Analysis Version 2 software (Biostat Inc, Englewood, NJ, USA). Forest plots were drawn using our own routines in Stata (Stata Corp, College Station, TX, USA)

### Assessment of heterogeneity

Clinical heterogeneity was explored through the narrative synthesis, identifying any variations in participants and interventions. Clinical heterogeneity was present in each meta-analysis owing to differences in the acupuncture techniques used and participant status (inpatient or outpatient) in the experimental interventions. Statistical heterogeneity was investigated for I^2^ statistics above 50%, i.e. demonstrative of moderate heterogeneity [9]. Secondary analyses based on variations in RCT characteristics were used to explore potential sources of heterogeneity.

## Results

### Description of included studies

#### Overview

**Fig. 1:**
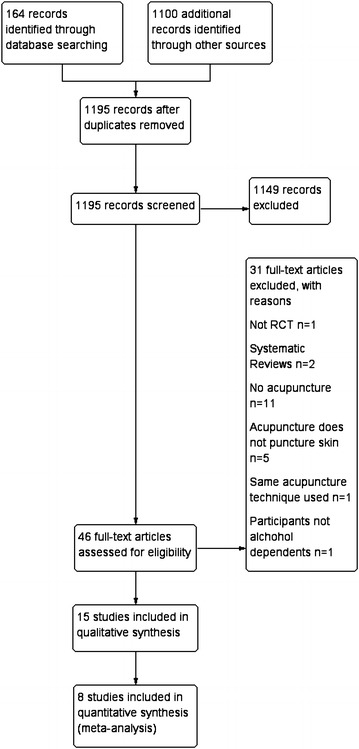
PRISMA flow diagram detailing the number of studies included and/or excluded at each stage

This updated review included 15 RCTs with a total of 1378 participants; Table 3 lists the characteristics of included RCTs. Figure 1 details the PRISMA flowchart of this review.

**Table 3:** Characteristics of included RCTs investigating the effect of acupuncture on alcohol dependence

First author, (year) location	Participants (total, status, inclusion criteria)	Study design; (allocation concealment^a^); (blinding^a^)	Intervention	Treatment frequency and duration	Treated acupoints	Control	Outcomes	Results reported	Adverse effects
Bullock (1987) USA [23]	N = 54 (males) Inpatients ≥20 admissions Previous treatment failure No identifiable support/group No full-time employment ≥6 months	2-arms; (B); (A)	AA + A	*Phase 1* daily (5 days) *Phase 2* 3 weekly (28 days) *Phase 3* 2 weekly (45 days)	Ear Lung, Shenmen, and either Liver, Kidney or occiput; LI4, SJ5. No manual or electro-stimulation	Non-specific ear points at “5 mml or less” from specific points	(1) Need for alcohol (2) Drinking episodes (3) Detox admissions (4) Craving (5) Completion rates	(1) *p* 0.003 (2) *p* 0.0076 (3) *p* 0.03 (4) *p* 0.015 (5) *p* 0.01	NR
Worner (1992) USA [12]	N = 56 (49 male) Outpatients Drinking within 10 days	3-arms; (B); (B)	AA + A	3 per week (3 months)	Ear Lung, Shenmen; LV3, ST36, SJ5, LI4, GV20	(1) Sham “transdermal stimulation” (electrocardiogram pads fixed to both forearms and to one lower leg), and TAU (2) TAU	(1) Completion rates (2) Relapse rates	No observed effects; *p* > 0.05	NR
Toteva (1996) Bulgaria [15]	N = 118 (103 male) Outpatients DSM-IV Abstinence from alcohol ≥10 days	2-arms; (B); (C)	A	Daily (12–15 sessions)	Five of six points drawn from: LI4, LI11, PC6, SJ5, SI4, GB8, GB14, HT7, Taiyang, Yintang	TAU	(1) Craving (2) Depressive symptoms (3) Participation in psychotherapeutic programs (4) Reduction in tremor (5) Remission rate	(1) *p* < 0.001 (2) *p* < 0.001 (3) *p* < 0.001 (4) *p* < 0.001 (5) *p* < 0.001	NR
Rampes (1997) England [10]	N = 59 (46 male) Outpatients DSM-III-R	3-arms; (A); (A)	AEA; square-wave continuous electric current of 100 Hz frequency	Weekly (6 weeks)	Ear Lung (AEA), Shenmen, Sympathetic Tone	(1) Knee 2, Internal Secretion (AEA), Elbow; and TAU (2) TAU	(1) Craving (VAS) (2) Units of alcohol (3) Breathalyser (4) Anxiety (5) Completion rates	(1) *p* 0.64 (2) *p* 0.69 (3) NS (4) *p* 0.16 (5) *p* 0.49 [vs Group 2]	Drowsiness, transient bleeding on removal of needles for 1 participant
Sapir-Weise (1999) Sweden [16]	N = 72 (51 male) Outpatients DSM-III-R	2-arms; (A); (A)	AA	*Phase 1* 5 weekly (2 weeks) *Phase 2* 3 weekly (4 weeks) *Phase 3* 2 weekly (4 weeks)	Ear Lung, Shenmen, Sympathetic; and TAU	AA non-specific 3–5 mm from specific points; and TAU	(1) Successful drinking pattern (2) Craving (3) Completion rates	(1) NS (2) NS (3) *p* 0.071	NR
Bullock (2002) USA [13]	N = 503 (253 male) Inpatients DSM-IV Anticipated stay ≥ 14 days	4-arms; (B); (A)	AA	Daily (excluding Sundays) for 3 weeks	Ear Liver, Lung, Shenmen, Sympathetic; TAU	(1) Symptom based, “acupuncturists were not constrained”; TAU (2) AA non-specific “within 5 mm” of specific points, TAU (3) TAU	(1) Craving (Likert Scale) (2) Completion rates	(1) *p* 0.024 (comparison of interventions) (2) *p* 0.06	NR
Karst (2002) Germany [20]	N = 34 (30 male) Inpatients ICD-10	2-arms; (B); (A)	AA + A	Daily (10 consecutive days)	Ear Kidney, Liver, Lung, Shenmen, Sympathetic; GV20, Extra 1, LI4; TAU	Sham acupuncture (Streitberger needle) at same specific points, TAU	(1) CIWA-Ar-Scale (2) BDI (3) EWL 60S (4) STAI	(1) *p* 0.045 (2) *p* 0.157 (3) NS (4) *p* 0.544	NR
Trümpler (2003) Switzerland [11]	N = 48 (28 male) Inpatients DSM-IV	3-arms; (B); (C)	AA	Daily (until end of withdrawal)	Needling “at ear points considered appropriate”; TAU	(1) Low level laser, TAU (2) Sham laser, TAU	(1) Duration of withdrawal (2) Duration of sedative prescription	(1) *p* 0.44 [vs Group 2] (2) NS	No local-side effects to acupuncture interventions
Kim (2005) Korea [14]	N = 22 (males) Inpatients Alcohol dependents	2-arms; (B); (A)	A	2 weekly (4 weeks)	Zùbin (K9)	Sham acupuncture (Park needle) at specific point	Craving (VAS)	*p* < 0.05	NR
Kunz (2007) Germany [19]	N = 109 (89 male) Inpatients ICD-10	2-arms; (B); (C)	AA	Daily (5 consecutive days)	Ear Kidney, Liver, Lung, Shenmen, Sympathetic; TAU	Aromatherapy, TAU	(1) AWS (2) Craving (VAS)	(1) NS (2) NS	Intervention: pain, mild bleeding, agitation; Control: negative thoughts, sore throat
Li (2009) China [24]	N = 100 (males) Inpatients CCMD-3	2-arms; (B); (B)	EA at a low frequency	Daily (for 6 weeks)	ST36 Nutritional supplement	TAU, nutritional supplement	(1) AWS (2) Relapse rate	(1) NS (2) *p* < 0.01	NR
Yao (2009) China [21]	N = 39 (males) Inpatients DSM-IV	2-arms; (B); (B)	A	Daily (5 consecutive days = 1 course) Total 1–3 courses	Sanyinjiao (SP-6), ST36, Insomnia	TAU, vitamins, antibiotics	(1) AWS (2) Clinical symptoms	(1) *p* < 0.05 (2) NR	Drowsiness for both groups, 1 control “addiction” to Western medicine
Zhang (2010) China [22]	N = 64 (males) Inpatients CCMD-3-R, HAMD-17	2-arms; (B); (B)	EA at a low frequency	Daily (for 4 weeks)	Baihui (DU20), Yingtang	Sham electro-acupuncture near specific points	(1) SCL-90 (2) HAM-D (3) HAM-A	(1) *p* < 0.05 (2) *p* < 0.05 (3) *p* < 0.05	NR
Lee (2015) Korea [18]	N = 20 (males) Inpatients DSM-IV	2-arms; (B); (A)	A	Twice a week (4 weeks)	Zùbin (KI9)	Sham acupuncture (Park needle) at specific point	Craving (VAS)	*p* < 0.01	NR
Tong (2015) China [17]	N = 80 (males) ICD-10	2-arms; (B); (B)	A, herbal medicine	Every other day (for 12 weeks)	Baihui	TAU	SF-36	*p* < 0.05	NR

*A* acupuncture, *AA* auricular acupuncture, *AEA* auricular electroacupuncture, *AWS* alcohol withdrawal syndrome, *BDI* Beck Depression Inventory, *CCMD-3-R* Criteria for Classification and Diagnosis of Mental Diseases, *CIWA-Ar-Scale* Clinical Institute Withdrawal Assessment for Alcohol, *DSM* Diagnostic and Statistical Manual of Mental Disorders, *EA* electroacupuncture, *EWL 60S* 60-item Adjective Checklist, *HAM-A* Hamilton Rating Scale for Anxiety, *HAM-D* Hamilton Rating Scale for Depression, *ICD-10* International Classification of Diseases, *NR* not reported, *NS* not significant, *SCL-90* symptom checklist-90, *STAI* stait-trait anxiety inventory, *TAU* treatment-as-usual, *VAS* Visual Analogue Scale

^a^Allocation concealment and blinding: (A) adequate, (B) unclear, (C) inadequate

#### Design

All of the studies were RCTs with a parallel study design. Most RCTs involved two arms, with the exception of three RCTs that were three-armed [10–12], and one that was four-armed [13].

#### Participants

Participants in 12 of the 15 RCTs satisfied one of three definitions of alcohol dependence: the Diagnostic and Statistical Manual of Mental Disorders (DSM)-III-R, the DSM-IV, the International Classification of Diseases (ICD-10), or the Chinese Classification of Mental Disorders (CCMD-3). However, three RCTs used additional selection criteria: one required participants to be inpatients of at least 14 days [13], one required participants to have been drinking within 10 days of enrolment in the outpatient alcoholism treatment programme [12], and one did not specify a definition, only stated that participants were alcohol-dependent [14]. The participants were inpatients in 11 of 15 RCTs, the exceptions were four RCTs involving outpatients [10, 12, 15, 16], and one RCT in which the setting was not specified [17]. The included RCTs were conducted in the USA (n = 3), Europe (n = 6), or East Asia (n = 6).

#### Description of acupuncture

The acupuncture techniques varied between RCTs. Eight RCTs used auricular acupuncture at points recommended by the UK National Acupuncture Detoxification Association (NADA); three of these eight RCTs also used body acupuncture. Seven RCTs used body acupuncture as the active treatment, either conventional needle acupuncture or electroacupuncture. All acupuncture techniques involved skin penetration.

The majority of RCTs (12 of 15) reported that the duration of needling was 30–45 min; two RCTs reported that the duration of needling was <30 min [15, 18], and one RCT did not report the duration of needling [14].

The duration of the treatment courses ranged from 5 days [19] to 12 weeks [17], or until a participant reached the withdrawal stage [11]. Six RCTs had fewer than 10 treatment sessions [10, 14, 18–21] two RCTs had 12–18 treatment sessions [13, 15], three RCTs had 24–30 treatment sessions [16, 22, 23], and three RCTs had 36–42 treatment sessions [12, 17, 24]. One RCT included a varying number of treatment sessions, as the sessions were continued until participants had become abstinent from alcohol [11].

Acupuncture was performed by an acupuncturist or Chinese medical practitioner (n = 7), a registered nurse-trained acupuncturist (n = 1), and NADA-trained mental health nurses (n = 1); six RCTs did not report these details. Five RCTs reported that interactions between the acupuncturist and participants were constrained by the study design; the majority (10 of 15 RCTs) did not report this information.

#### Description of controls

Ten RCTs used sham acupuncture as a control, with the aim of minimising differences in the treatment experience between groups, with participants blinded to the intervention. Fourteen RCTs used treatment-as-usual as an intervention, either as a control group (n = 5) or as an adjunct to the experimental treatment (n = 9). In total there were 20 control groups.

#### Outcomes

Alcohol craving and alcohol withdrawal symptoms were measured at key time points in the RCTs: at baseline, during intervention, and at follow-up. Visual Analogue Scale and Likert-scaled options were used to assess craving. The Alcohol Withdrawal Syndrome Scale, the Mainz Alcohol Withdrawal Scale, and the Clinical Institute Withdrawal Assessment were used to assess the severity of withdrawal symptoms. Where data on adverse events were reported, they were collected through observation by acupuncturists or self-reported by participants.

#### Cochrane Risk of Bias

**Fig. 2:**
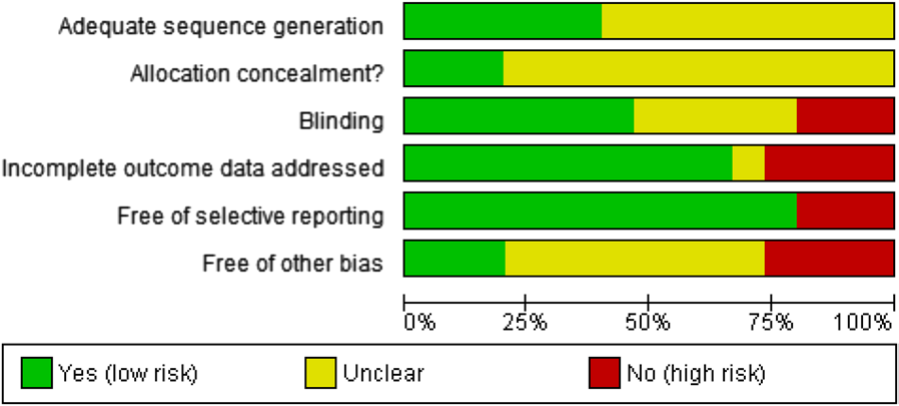
Risk of Bias: review authors’ judgements about each methodological quality item presented as percentage across all studies

**Fig. 3:**
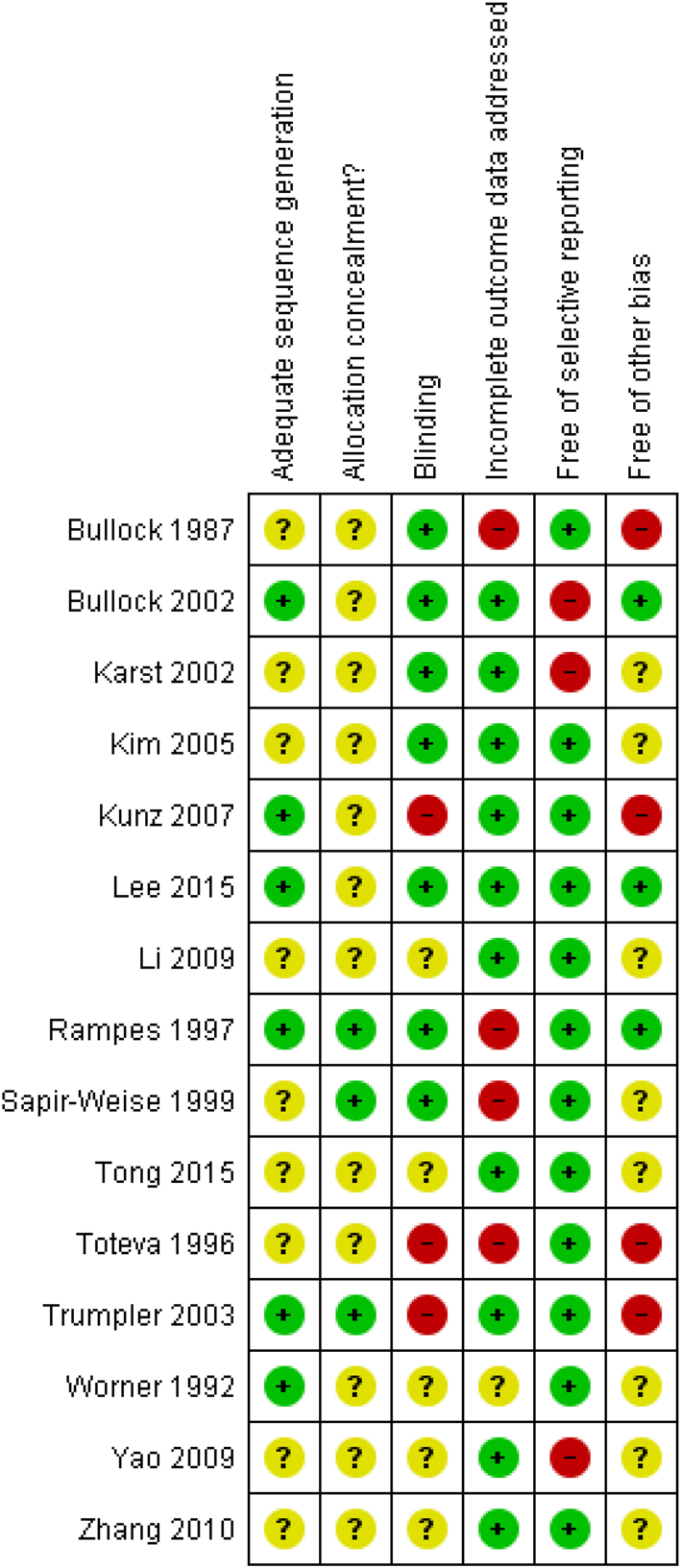
Risk of bias: review authors’ judgements for each domain

Assessments using the Cochrane Risk of Bias tool [7] are presented in Figs. 2 and 3. Details by RCT are provided in Appendix S2 (Additional file 1).

#### Jadad Scale

The Jadad Scale [8] scores are presented in Table 4. Five RCTs had a high methodological quality score (≥3) and ten RCTs had a low methodological quality score (≤2). Owing to the difficulties in blinding the acupuncturist, none of the RCTs were able to score the maximum of five points; therefore a score of three, which is classified as high methodological quality, was the maximum score possible.

**Table 4:** Jadad Scale representing scores in descending order

Study	Described as RCT?	Adequate randomisation?	Double-blind?	Details of double-blinding?	Reasons stated for withdrawals?	Total score
Bullock et al. [13]	+1	+1	+0	+0	+1	3
Kunz et al. [19]	+1	+1	+0	+0	+1	3
Lee et al. [18]	+1	+1	+0	+0	+1	3
Rampes et al. [10]	+1	+1	+0	+0	+1	3
Trümpler et al. [11]	+1	+1	+0	+0	+1	3
Worner et al. [12]	+1	+1	+0	+0	+0	2
Karst et al. [20]	+1	−1	+0	+0	+1	1
Kim et al. [14]	+1	−1	+0	+0	+1	1
Li and Guo [24]	+1	−1	+0	+0	+1	1
Yao [21]	+1	−1	+0	+0	+1	1
Zhang et al. [22]	+1	−1	+0	+0	+1	1
Bullock et al. [23]	+1	−1	+0	+0	+0	0
Sapir-Weise et al. [16]	+1	−1	+0	+0	+0	0
Toteva and Milanov [15]	+1	−1	+0	+0	+0	0
Tong et al. [17]	+1	−1	+0	+0	+0	0

#### Primary meta-analyses

##### Craving

**Fig. 4:**
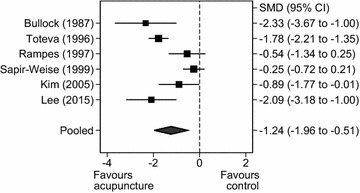
*Forest plot*: acupuncture versus all controls (combined sham and treatment as usual) for alcohol craving

Nine RCTs assessed alcohol craving. One RCT compared acupuncture with an active treatment (aromatherapy) and was therefore excluded from the analysis as it was considered an equivalent intervention [19]. Only six RCTs [10, 14–16, 18, 23] (n = 345 participants) provided sufficient statistical information to include in the meta-analysis. Five of these RCTs measured craving using a quantitative scale; however, only one RCT provided the odds ratio [23]. Therefore, the odds ratio was converted into the standardised difference, using a method suggested by Borenstein et al. [25], so that the statistical information could be included in the analysis. There was evidence of a statistically significant effect of acupuncture on self-reported experience of craving compared to all control groups (sham and treatment-as-usual) (SMD = −1.24, 95% CI = −1.96 to −0.51, Q = 30.89, df = 5, *P* < 0.001, I^2^ = 83.8%; Fig. 4).

##### Alcohol withdrawal

**Fig. 5:**
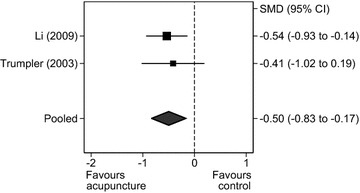
*Forest plot*: acupuncture versus all controls for withdrawal symptoms

Five RCTs assessed alcohol withdrawal symptoms. Again, the one RCT that compared acupuncture with an active treatment (aromatherapy) was excluded from the meta-analysis [19]. Two further RCTs were excluded as they provided insufficient statistical data [20, 21]. From the remaining two RCTs [11, 24] (n = 148 participants), there was evidence of a significant effect of acupuncture compared to all controls for alcohol withdrawal symptoms (SMD = −0.50, 95% CI = −0.83 to −0.17, Q = 0.11, df = 1, *P* = 0.003, I^2^ = 0%; Fig. 5).

#### Secondary subgroup and sensitivity meta-analyses

For alcohol craving, the data were split to conduct analyses that might lead to an explanation of the heterogeneity.

##### Acupuncture versus sham acupuncture for alcohol craving

**Fig. 6:**
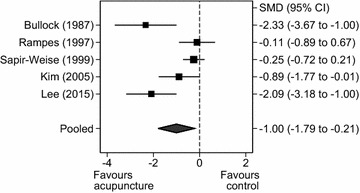
*Forest plot*: acupuncture versus sham acupuncture for craving

Pooling only those RCTs that compared acupuncture with sham acupuncture (n = 211 participants), acupuncture significantly reduced self-reported experience of craving (SMD = −1.00, 95% CI = −1.79 to −0.21, Q = 17.74, df = 4, *P* = 0.013, I^2^ = 77.4%; Fig. 6). There was a small reduction in the I^2^ statistic.

##### Studies conducted in Western countries for alcohol craving

**Fig. 7:**
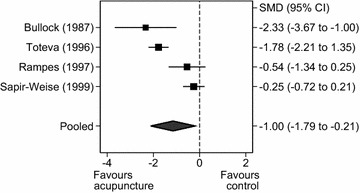
*Forest plot*: acupuncture versus control groups for craving in Western studies

Pooling the endpoint values for the Western-based RCTs (n = 303 participants), there was a statistically significant effect of acupuncture on self-reported experience of craving (SMD = −1.15, 95% CI = −2.12 to −0.18, Q = 27.44, df = 3, *P* = 0.02), with slightly increased heterogeneity (I^2^ = 89.07%; Fig. 7).

##### Male-only randomised controlled trials for alcohol craving

**Fig. 8:**
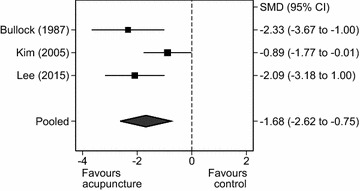
*Forest plot graph*: acupuncture versus control groups for craving in male participants

Out of the 15 RCTs (n = 1378 participants), eight RCTs included females (n = 327 participants) and seven RCTs were male-only studies. An analysis was conducted on three of these male-only RCTs. Pooling the endpoint values, there was a statistically significant effect of acupuncture versus controls on self-reported experience of craving (SMD = −1.68, 95% CI = −2.62 to −0.75, Q = 4.46, df = 2, *P* < 0.001, I^2^ = 55.12%; Fig. 8). Excluding the RCTs containing both male and female participants considerably reduced the heterogeneity.

#### Adverse events

Four RCTs reported adverse effects, although data were limited. Kunz et al. [19] reported pain, mild bleeding on insertion of needles, and agitation in the acupuncture group, while the aromatherapy group experienced agitation, sneezing, negative thoughts, and sore throat. Yao [21] reported that drowsiness was experienced in both the acupuncture group (n = 1) and the control group (n = 4); however, one participant in the control group also reported an addiction to pharmaceutical medicine. Rampes et al. [10] reported drowsiness and transient bleeding on removal of needles in the electroacupuncture group at specific acupuncture points; one participant in the non-specific electroacupuncture group dropped out of the RCT owing to pain. Trümpler et al. [11] reported that no local side effects were observed. However, one participant in the acupuncture group experienced self-limiting generalised convulsions whilst sleeping on the fifth day; the convulsions lasted 5 min, and were judged to be a withdrawal-related epileptic seizure on clinical grounds.

## Discussion

### Major findings

Our review comprised 15 RCTs with 1378 patients from eight countries published in either Chinese or English. Our findings provide the first clear evidence that acupuncture might be considered an effective intervention for alcohol dependence. This evidence of significant effectiveness was observed in our two primary analyses, one showing a reduction in alcohol craving and one showing a reduction in withdrawal symptoms. In all three of our secondary analyses, we found significant reductions in alcohol craving: comparing acupuncture versus sham acupuncture; comparing acupuncture with controls in only those RCTs conducted in Western countries; and in those RCTs that recruited males only. We note that the effect size of acupuncture when all RCTs are combined is slightly larger than when compared with sham acupuncture RCTs or with Western-based RCTs, and is slightly smaller when compared with male-only RCTs. While the overall effect estimate favoured acupuncture for all analyses, some of the individual RCTs found non-significant effects of acupuncture on alcohol dependence. By pooling the data across RCTs, we increased the overall power, which led to the conclusion that acupuncture was an effective intervention.

The present review has identified pre-specified secondary analyses to explore reasons for potential heterogeneity and aid interpretation. The I^2^ value for acupuncture versus all controls for alcohol craving was 83.8%; this value reduced slightly for the analysis of acupuncture versus sham acupuncture in alcohol craving (77.4%). An analysis of alcohol craving in Western-based RCTs showed increased heterogeneity (89.1%). An analysis of acupuncture for male-only participants for alcohol craving substantially reduced the heterogeneity (55.1%). Interpreting these values with reference to the 50% threshold [9] indicates that the results must be treated with caution.

### Strengths and limitations

The included RCTs were rigorously assessed using two quality assessments. The included RCTs were not all of a high standard, with 10 of the 15 RCTs scoring two or less on the Jadad scale. Moreover, only eight RCTs contained sufficient statistical information on outcomes for meta-analysis; this limited the scope for comparing acupuncture with some controls. For example, because of a lack of sufficient quantitative outcome data we were unable compare acupuncture with treatment-as-usual. While we have combined changes in continuous outcomes using standardised mean differences for the purposes of conducting the meta-analyses, the individual measures of craving and withdrawal symptoms may have different measurement characteristics, which would lead to an increase in heterogeneity. Furthermore, most of the included RCTs contained a small sample size. These factors resulted in a small number of participants being included in the final analyses. The present review did not include a sufficient number of RCTs to address publication bias.

The majority of the included RCTs were conducted in a hospital setting; therefore, generalisation of the results to community settings must be done with caution. The clinical heterogeneity associated with the different acupuncture techniques limits our ability to identify which technique was more effective for treatment of alcohol disorders. Acupuncture treatments also varied in duration, frequency, and the acupuncture points used, making it difficult to assess the key characteristics that might be associated with the effectiveness of the intervention. Whilst some RCTs combined auricular acupuncture and body acupuncture as part of the same treatment, most RCTs involved either auricular or body acupuncture. Given the potential variation in modes of action, combining these two modalities (either within RCTs or between RCTs), will add to the clinical heterogeneity. Sham acupuncture also varied across RCTs. Moreover, sham acupuncture cannot be considered a physiologically inert intervention, thereby potentially leading to an underestimate of the effect of the acupuncture.

### Comparisons with previous systematic reviews

The present review included an increased number of RCTs (n = 15) compared with the previous reviews of Kunz et al. [5] (n = 6) and Cho and Whang [6] (n = 11). Kunz et al. [5] did not conduct a meta-analysis, and reported that there was insufficient evidence to conclude whether auricular acupuncture is an effective intervention in the treatment of alcohol and substance abuse. Cho and Whang conducted a meta-analysis of acupuncture versus sham acupuncture for treatment completion rates, reporting no significant differences [6] but no meta-analysis was conducted by them for craving because of insufficient data. By contrast, our study found sufficient data to conduct a meta-analysis of craving with six trials included [10, 14–16, 18, 23]. Moreover, the present review is the first to provide a meta-analysis of withdrawal symptoms, albeit based on only two RCTs [11, 24]. The results we present here therefore represent an update of the evidence, with the caveat that our results are based on a small number of RCTs with variable risks of bias.

### Implications for research and practice

Non-pharmacological treatments are an important therapeutic option for people with alcohol-related problems, and there is some evidence that acupuncture is valued by service users [2]. Recommendations for future studies to improve the quality and statistical power of the next review on this topic include:

- *Larger sample sizes* Further RCTs with larger sample sizes are needed. An increased sample size increases power statistically, enhancing a meta-analysis. Large sample sizes also provide the opportunity to explore potential moderators and mediators of response.
- *Relevant statistical information reported* Summary statistics on outcomes should be reported to a high standard so that these data can be included, thereby enhancing the robustness of the meta-analyses. An assessment for publication bias could also be conducted using a funnel plot.
- *Female participants* More studies should recruit female as well as male participants, so that results can be better generalised to the female population of problem drinkers.
- *Longer-term follow ups* for at least 1 year are recommended, along with parallel cost-effectiveness analyses.
- *Increase methodological quality* Future studies should follow STRICTA recommendations [26], which are guidelines specifically aimed at producing high quality reporting of acupuncture interventions, and the Consolidated Standards of Reporting Trials (CONSORT) statement [27], which are guidelines on study design and reporting to reduce potential bias and increase methodological quality.

## Conclusion

This study showed that acupuncture was potentially effective in reducing alcohol craving and withdrawal symptoms and could be considered as an additional treatment choice and/or referral option within national healthcare systems.

## Additional file

**Additional file 1.** Search strategy and risk of bias tables.

## Abbreviations

A: acupuncture
AA: auricular acupuncture
AEA: auricular electroacupuncture
AMED: allied and complementary medicine database
CCMD: Chinese Classification of Mental Disorders
CI: confidence interval
CNKI: China National Knowledge Infrastructure
CONSORT: consolidated standards of reporting trials
df: degrees of freedom
DSM: Diagnostic and Statistical Manual of Mental Disorders
EA: electroacupuncture
EMBASE: excerpta medica database
ICD: International Classification of Diseases
I^2^: heterogeneity statistic
MEDLINE: national library of medicine database
MeSH: medical subject headings
NADA: national acupuncture detoxification association
NICE: national institute for health and care excellence
NR: not reported
NS: not significant
OVID: OVID database search interface
PsycINFO: psychology information database
PRISMA: preferred reporting items for systematic reviews and meta-analyses
PubMed: search engine for national library of medicine databases
Q: heterogeneity statistic
RCT: randomised controlled trial
Sino-med: Chinese biomedical literature service system
SMD: standardised mean difference
STRICTA: revised standards for reporting interventions in clinical trials of acupuncture
TAU: treatment-as-usual
VIP: Chinese science and technology periodicals database
WanFang: China medical collections database
UK: United Kingdom
