# Supplementary material for: Acupuncture as an intervention to reduce alcohol dependency: a systematic review and meta-analysis
Source: Chin Med. 2016 Dec 15;11:49. doi: 10.1186/s13020-016-0119-4 (PMC5160025; doi:10.1186/s13020-016-0119-4)

Additional file 1

**Appendix S1: MEDLINE (OvidSP) Search Strategy**

This search will be based on the following MeSH terms and keywords:

Search strategy to locate alcohol dependence:

1. alcohol related disorders.mp. or exp Alcoholism/ or exp Alcohol-Related Disorders/ (99745)

2. exp Alcohol Drinking/ or drinking behaviour.mp. or exp Drinking Behavior/ (60216)

3. exp Alcohol Withdrawal Delirium/ or exp Substance Withdrawal Syndrome/ or alcohol withdrawal.mp. (21750)

4. alcohol dependence.mp. (7160)

5. exp Substance-Related Disorders/ or alcohol dependent.mp. or exp Substance Withdrawal Syndrome/ (234093)

6. alcohol abuse.mp. (11498)

7. alcohol abstinence.mp. or exp Alcohol Abstinence/ (662)

8. alcohol addiction.mp. (803)

9. alcohol misuse.mp. or exp Alcohol-Related Disorders/ (100170)

10. alcohol abstinence.mp. or exp Alcohol Abstinence/ (662)

11. alcohol craving.mp. (504)

12. exp Alcohol Drinking/ or alcohol$ beverage$.mp. or exp Alcoholic Beverages/ (67210)

13. exp Alcoholic Intoxication/ or alcohol intoxication.mp. (12292)

14. substance related disorder.mp. or exp Substance-Related Disorders/ (233911)

1. 1 or 2 or 3 or 4 or 5 or 6 or 7 or 8 or 9 or 10 or 11 or 12 or 13 or 14 (291208)

Search strategy to locate acupuncture:

16. exp Acupuncture Analgesia/ or exp Acupuncture Therapy/ or exp Acupuncture/ or exp Acupuncture Points/ or exp Acupuncture, Ear/ or acupuncture.mp. (20219)

17. auricular acupuncture.mp. (221)

18. electroacupuncture.mp. or exp Electroacupuncture/ (3318)

19. body acupuncture.mp. (107)

20. medical acupuncture.mp. (54)

21. traditional acupuncture.mp. (167)

22. traditional Chinese medicine.mp. or exp Medicine, Chinese Traditional/ (17648)

23. alternative therapy.mp. or exp Complementary Therapies/ (186552)

1. 16 or 17 or 18 or 19 or 20 or 21 or 22 or 23 (191988)

Search strategy to locate human studies:

- Humans/ (14029583)

Search strategy to locate RCTs:

26. exp Randomized Controlled Trials as Topic/ or RCT.mp. (105076)

27. 15 and 24 and 25 and 26 (35)

Appendix S2 – Risk of Bias Tables

Bullock[23]


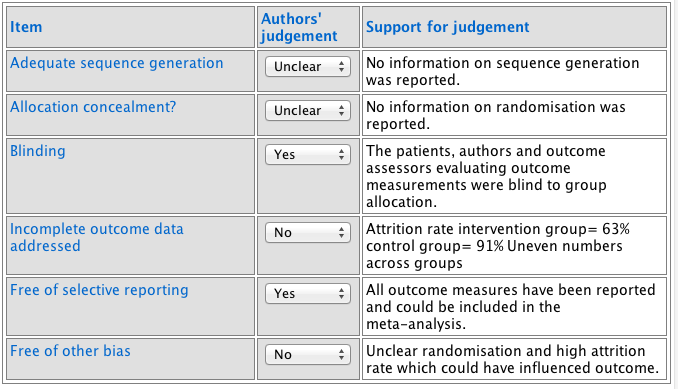


Bullock[13]


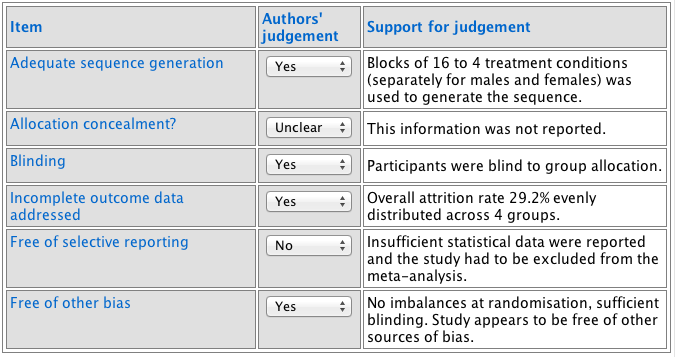


Karst[20]


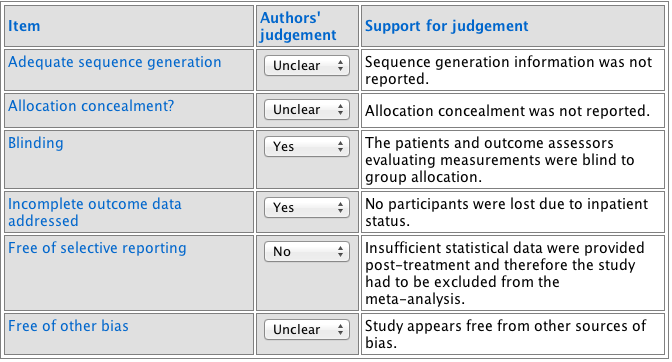


Kim[14]


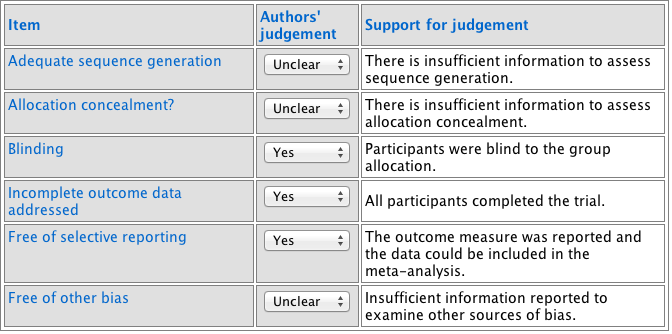


Kunz[19]


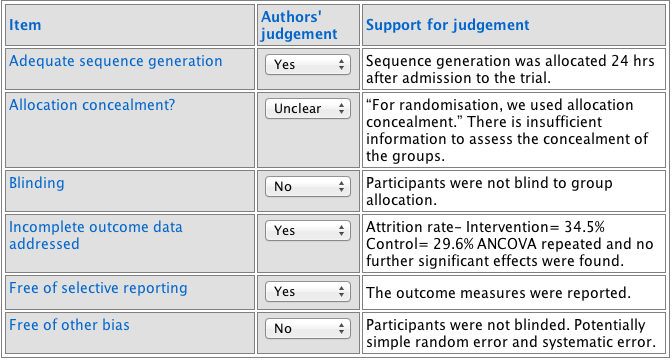


Lee[18]


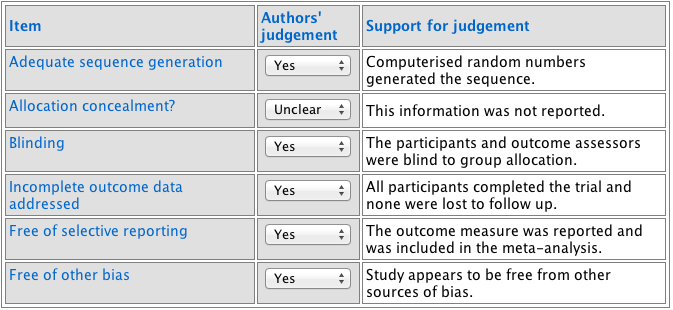


Li[24]


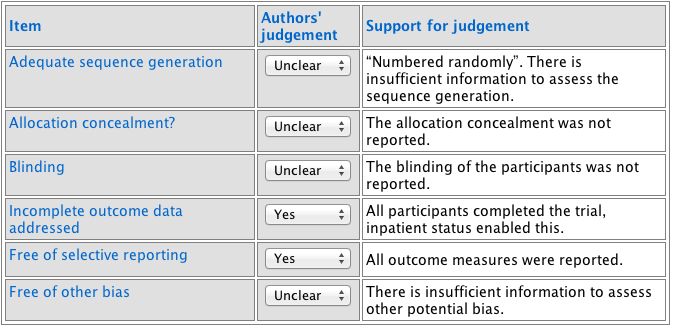


Rampes[10]


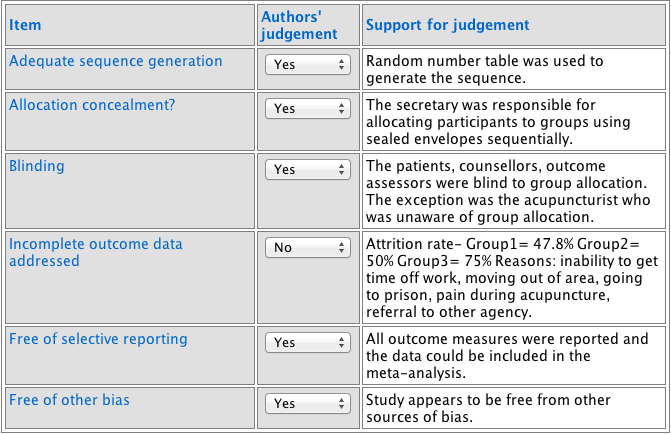


Sapir-Weise[16]
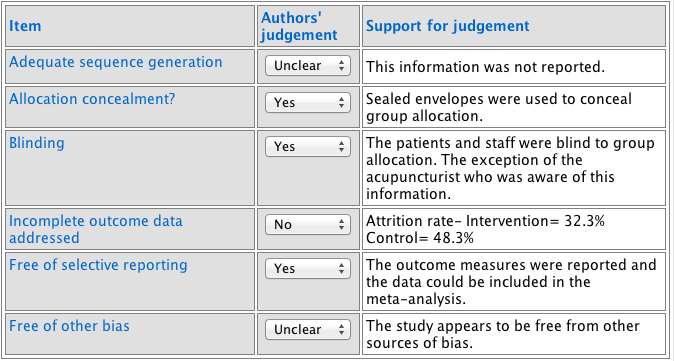


Tong[17]


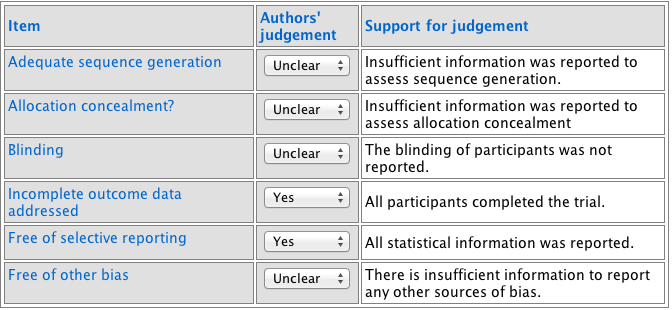


Toteva[15]


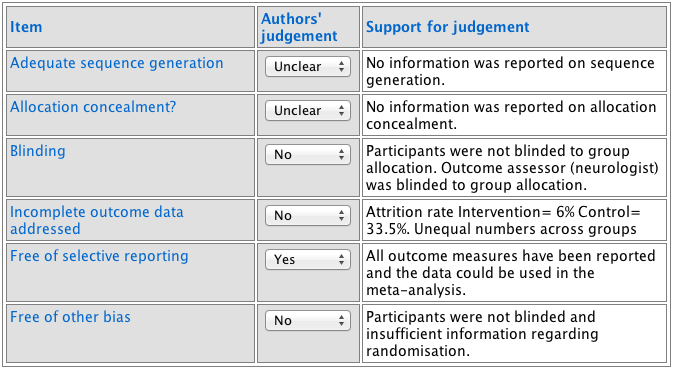


Trumpler[11]


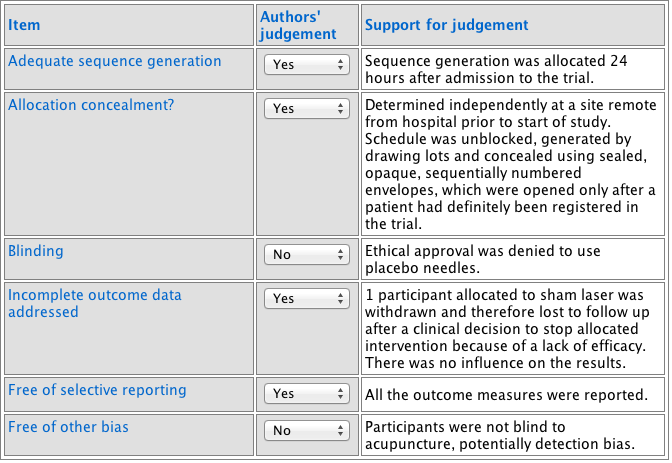


Worner[12]


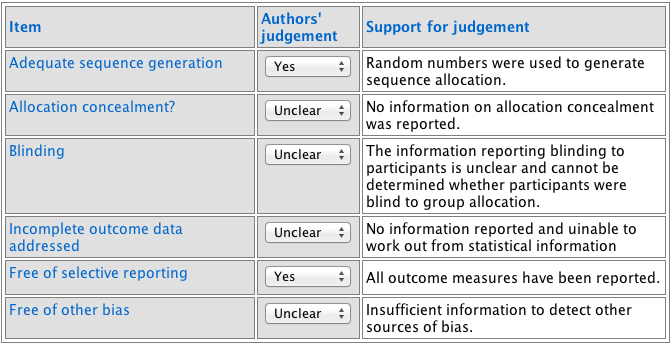


Yao[21]


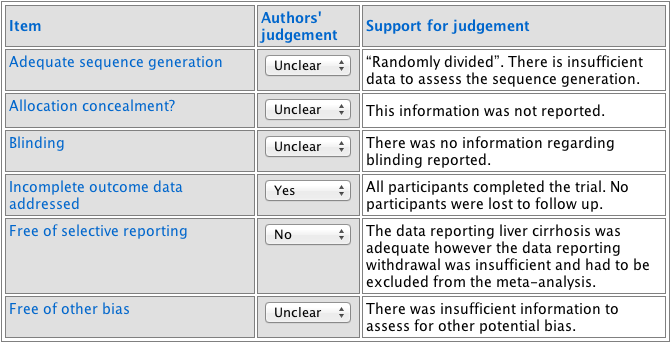


Zhang[22]


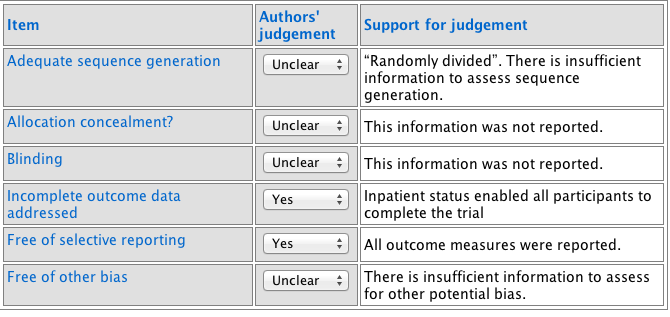

Supplement: Supplementary file 1 — Additional file 1. Search strategy and risk of bias tables. [file 13020_2016_119_MOESM1_ESM.docx]
